# Individualized atomoxetine response and tolerability in children with ADHD receiving different dosage regimens: the need for *CYP2D6* genotyping and therapeutic drug monitoring to dance together

**DOI:** 10.1038/s41398-024-02859-2

**Published:** 2024-03-19

**Authors:** Hong-Li Guo, Dan-Dan Wu, Di Fu, Yue Li, Jie Wang, Yuan-Yuan Zhang, Wei-Jun Wang, Jian Huang, Wei-Rong Fang, Jing Xu, Ya-Hui Hu, Qian-Qi Liu, Feng Chen

**Affiliations:** 1 Department of Pharmacy, Children’s Hospital of Nanjing Medical University, Nanjing, China; 2 Department of Children Health Care, Children’s Hospital of Nanjing Medical University, Nanjing, China; 3 School of Basic Medicine and Clinical Pharmacy, China Pharmaceutical University, Nanjing, China; 4 Present Address: Visiting graduate student from China Pharmaceutical University, Nanjing, China

**Keywords:** ADHD, Diseases

## Abstract

Integrating *CYP2D6* genotyping and therapeutic drug monitoring (TDM) is crucial for guiding individualized atomoxetine therapy in children with attention-deficit/hyperactivity disorder (ADHD). The aim of this retrospective study was (1) to investigate the link between the efficacy and tolerability of atomoxetine in children with ADHD and plasma atomoxetine concentrations based on their *CYP2D6* genotypes; (2) to offer TDM reference range recommendations for atomoxetine based on the *CYP2D6* genotypes of children receiving different dosage regimens. This retrospective study covered children and adolescents with ADHD between the ages of 6 and <18, who visited the psychological and behavioral clinic of Children’s Hospital of Nanjing Medical University from June 1, 2021, to January 31, 2023. The demographic information and laboratory examination data, including *CYP2D6* genotype tests and routine TDM of atomoxetine were obtained from the hospital information system. We used univariate analysis, Mann-Whitney U nonparametric test, Kruskal-Wallis test, and the receiver operating characteristic (ROC) curve to investigate outcomes of interest. 515 plasma atomoxetine concentrations of 385 children (325 boys and 60 girls) with ADHD between 6 and 16 years of age were included for statistical analysis in this study. Based on genotyping results, >60% of enrolled children belonged to the CYP2D6 extensive metabolizer (EM), while <40% fell into the intermediate metabolizer (IM). CYP2D6 IMs exhibited higher dose-corrected plasma atomoxetine concentrations by 1.4-2.2 folds than those CYP2D6 EMs. Moreover, CYP2D6 IMs exhibited a higher response rate compare to EMs (93.55% vs 85.71%, *P* = 0.0132), with higher peak plasma atomoxetine concentrations by 1.67 times than those of EMs. Further ROC analysis revealed that individuals under once daily in the morning (*q.m*.) dosing regimen exhibited a more effective response to atomoxetine when their levels were ≥ 268 ng/mL (AUC = 0.710, *P* < 0.001). In addition, CYP2D6 IMs receiving *q.m*. dosing of atomoxetine were more likely to experience adverse reactions in the central nervous system and gastrointestinal system when plasma atomoxetine concentrations reach 465 and 509 ng/mL, respectively. The findings in this study provided promising treatment strategy for Chinese children with ADHD based on their *CYP2D6* genotypes and plasma atomoxetine concentration monitoring. A peak plasma atomoxetine concentration higher than 268 ng/mL might be requisite for *q.m*. dosing. Assuredly, to validate and reinforce these initial findings, it is necessary to collect further data in controlled studies with a larger sample size.

## Introduction

Attention deficit/hyperactivity disorder (ADHD) is a neurodevelopmental disorder characterized by excessive amounts of inattention, hyperactivity, and impulsivity that are pervasive, impairing in multiple contexts, and otherwise age-inappropriate [1–3]. A recent meta-analysis reported a pooled ADHD prevalence of 7.2% (95% CI: 6.7–7.8%) among children in the world [4]. Similarly, the overall pooled ADHD prevalence in China was 6.26% (95% CI: 5.36–7.22%) [5], suggesting that ADHD affects approximately 23 million Chinese children and adolescents. In addition, ADHD is diagnosed approximately twice as often in boys [14.0% (95% CI, 13.1-15.0%)] than in girls [6.3% (95% CI, 5.6-7.0%)] [6]. Of note, about 30–50%, even up to 70%, of people diagnosed in childhood continue to have ADHD in adulthood, with 2.58% of adults estimated to have ADHD which began in childhood [7–9]. ADHD can elevate the likelihood of other psychiatric disorders, academic and professional underachievement, incidents, involvement in criminal activities, impaired social functioning, and substance dependencies over the course of a person’s life [8].

ADHD management typically involves medications or counseling, either alone or both. Medications approved by the FDA comprise stimulants (like amphetamines and methylphenidate), generally recommended as the first-line pharmacologic treatment, and nonstimulants (like atomoxetine and extended-release clonidine and guanfacine), always taken as the second-line therapy [2]. However, the 2014 Japanese clinical guidelines and the 2015 Chinese guidelines recommend both the nonstimulants and the stimulants as first-line pharmacological treatment for children and adolescents with ADHD [10].

Indeed, atomoxetine was approved by the FDA to treat ADHD on July 27, 2002 and was subsequently introduced in China on September 28, 2007. In clinical settings, however, significant variability in the clinical efficacy, tolerability, and pharmacokinetics of atomoxetine is a pestering problem that must be confronted in the ADHD management [11–14]. The authors have also observed many sobering phenomena during routine plasma monitoring of atomoxetine. For example, some children achieved higher exposure to atomoxetine at very low doses, while others experienced the opposite—high doses but low systemic exposure; some pediatric patients tolerated atomoxetine poorly at low exposure, while others tolerated it well even at high drug concentrations. In another scenario, some other children experienced low doses and low exposures, tolerated the drug very well but showed poor clinical efficacy. In these cases, alternative medications were chosen instead of adjusting the dosage regimen [10].

Individualized dosing strategy may be beneficial for addressing above clinical concerns. However, the reality is that there have been very few precision medicine studies focusing on atomoxetine conducted in the past 15 years in China specifically for pediatric ADHD patients. Meanwhile, Chinese guidelines and expert consensus do not involve recommendations or specific descriptions of personalized medication [15]. In contrast, guidelines published worldwide, like Clinical Pharmacogenetics Implementation Consortium (CPIC) guideline and Dutch Pharmacogenetics Working Group (DPWG) guideline, have provided individual descriptions of personalized dosing strategies for atomoxetine, which have played a crucial role in enhancing efficacy and improving tolerability [16–18]. These guidelines specifically emphasize the clinical value of *CYP2D6* genetic polymorphism testing, since CYP2D6 is the main metabolizing enzyme of atomoxetine, and therapeutic drug monitoring (TDM) in guiding the implementation of personalized therapy with the nonstimulant.

Therefore, the aim of this retrospective study was (1) to explore the association between the effectiveness and tolerability of atomoxetine in treating children with ADHD and plasma atomoxetine concentrations; (2) to provide potentially matched TDM reference range recommendations for atomoxetine in children receiving different dosing regimens based on their *CYP2D6* genotypes. This study focuses on striving for personalized medication strategies for atomoxetine therapy in children with ADHD.

## Materials and Methods

### Study population

**Fig. 1:**
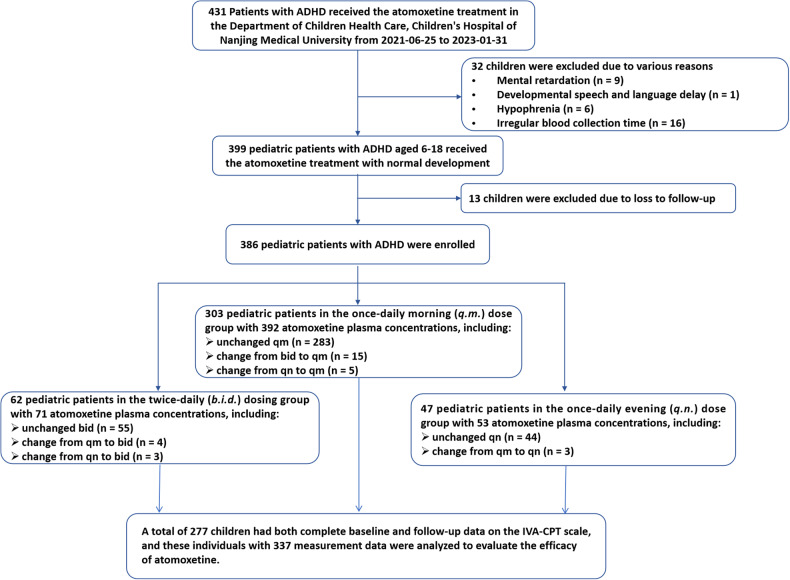
Inclusion and exclusion processes for study subjects. A total of 386 children and adolescents were enrolled in this study and 277 eligible participants were analyzed to evaluate the efficacy.

This retrospective study covered children and adolescents between the ages of 6 and <18, who visited the psychological and behavioral clinic of Children’s Hospital of Nanjing Medical University from June 1, 2021, to January 31, 2023. All participants had a confirmed ADHD diagnosis according to the Diagnostic and Statistical Manual of Mental Disorders (DSM)-IV criteria. All children had received atomoxetine (longer than 4 weeks) treatment and underwent routine TDM. The exclusion criteria were as follows: (1) children who have been diagnosed with mental retardation, pervasive developmental disorders, delayed language development, developmental disorders in special learning skills, childhood schizophrenia, bipolar disorder; (2) individuals with intellectual deficiency or low intelligence as determined by the Weschler/Raven intelligence test for children; (3) those who failed to provide blood samples within the designated time frame; and (4) individuals who were lost to follow-up. The inclusion and exclusion criteria are listed in Fig. 1.

### Clinical data collection

The routine TDM data were collected at the Pharmaceutical Sciences Research Center in the same hospital. Sample information was extracted from the standard TDM requisition forms (telephone follow-up), including concomitant medication, time interval between last dosing and sampling, and daily dosage. The recommended sampling time interval for atomoxetine is 1.5–4 h, in concordance with the CPIC guideline [17].

Furthermore, additional clinical data were obtained from the hospital information system (HIS), including (1) demographic information of the children and adolescents enrolled; (2) their ADHD history, response to atomoxetine therapy, and records of adverse reactions; (3) duration and dosage of drug usage; (4) monitoring data of atomoxetine concentration; (5) *CYP2D6* genotype data.

### Atomoxetine treatment regimens

All eligible children and adolescents were administered standard doses of atomoxetine hydrochloride, starting at 10 mg/day once daily in the morning (*q.m*.) for 1 week and 25 mg/day *q.m*. for the second week. Depending on the clinical efficacy, tolerability, and body weight (BW) of the children, the dosage was adjusted to 1.2–1.4 mg/(kg·d) within 2-4 weeks of administration, and the maximum dose did not exceed 1.4 mg/(kg·d) to maintain atomoxetine therapy. Based on the patient’s tolerance, the medication regimen also will be adjusted to either twice daily (*b.i.d*.) or once daily at night (*q.n*.).

### Definitions

According to the frequency of medication following the atomoxetine treatment, the enrolled children and adolescents with ADHD were categorized into three regimens: *q.m*., *b.i.d*., and *q.n*. Additionally, based on the age at enrollment, they fell into two categories: school-age (6 to <12 years old) and adolescence (12 to <18 years old). The clinical response to atomoxetine was described as “responsive” or “non-responsive” by the treating physicians combining the results of determination and evaluation using the DSM-IV and the Integrated Visual and Auditory Continuous Performance Test (IVA-CPT). Briefly, IVA-CPT scale can be used to evaluate hyperactivity-impulsiveness and attention deficit by taking into account both omission errors and commission errors. This scale includes 3 auditory area quotients (*i.e*., auditory, visual, and full-scale response control quotient) and 3 visual area quotients (*i.e.*, auditory, visual, and full-scale attention quotient) in each item. An improvement in the score for any of these quotients is considered as “responsive”. Furthermore, if there is a significant decrease in DSM-IV scores compared to the baseline and positive changes are observed during interviews with physician, parents, and the children, the children are also labeled as “responsive” even if there are no significant changes observed in the IVA-CPT score. A standardized questionnaire for a more precise assessment did not exist.

The adverse reactions associated with atomoxetine were classified into gastrointestinal reactions and reactions related to the central nervous system. Gastrointestinal reactions included decreased appetite, abdominal discomfort, nausea and vomiting, indigestion, and constipation. Neurological adverse reactions included drowsiness, easily irritated, low mood, dizziness and headache, easy to wake up, and difficulty falling asleep. In addition, the sampling time for TDM implementation was selected to coincide with primary therapeutic effect and adverse effect.

### Routine TDM of atomoxetine

In accordance with the current clinical requirements for TDM, venous blood samples were collected into ethylenediaminetetraacetic acid anticoagulant tubes at the 2-h time point after administration in the *q.m*. and *b.i.d*. groups, whereas at the 12-h time point after administration in the *q.n*. group. Whole blood samples are routinely transported to our lab for monitoring steady-state plasma atomoxetine levels in pediatric patients with ADHD. These samples were centrifuged at 4000 rpm for 8 minutes. The plasma was then utilized to determine the level of atomoxetine.

Analytical assays were performed as described in detail by our lab [19]. In brief, the liquid chromatography (LC) - tandem mass spectrometry (MS) system consisted of a Triple Quad^TM^ 4500MD MS (AB Sciex Pte. Ltd, Singapore) interfaced via a Turbo V^TM^ ion source with a Jasper^TM^ LC system (AB Sciex Pte. Ltd, Singapore), which comprises a binary pump (Sciex Dx^TM^), an online degasser (Sciex Dx^TM^), an autosampler (Sciex Dx^TM^), and a column oven (Sciex Dx^TM^). The AB-SCIEX Analyst software packages (Version 1.6.3) were used to control the LC-MS/MS system, as well as for data acquisition and processing.

The chromatographic separation was achieved on a Kinetex C_18_ column (2.1 × 50 mm, 2.6 μm, Phenomenex) with a security Guard-C18 column (4 × 2.0 mm, Phenomenex), pumped at a flow rate of 0.25 mL/min. Gradient elution was carried out with mobile phase A (PhA) consisting of 5 mM ammonium acetate (NH_4_Ac) and 0.1 mM formic acid (FA) in water and mobile phase B (PhB) of methanol (MeOH) containing the same NH_4_Ac and FA levels. A gradient program was used as follows: 0–2.0 min, 58% PhB; 2.0–2.1 min, from 58 to 100% PhB; 2.1–3.5 min, 100% PhB; 3.5–3.6 min, from 100 to 58% PhB; 3.6–5 min, 58% PhB. The temperature for column and autosampler were kept at 40 °C and 4 °C, respectively. MeOH precipitation method was used for sample clean-up and the 5 μL supernatant was injected into the LC-MS/MS for analysis. Ionization mode was positive electrospray ionization (ESI) and two precursor-product ion pairs, m/z 256.4 → 43.8 and 259.3 → 47.0, were monitored for atomoxetine and its internal standard, respectively. Atomoxetine quantification was normalized by using stable-isotope-labeled atomoxetine-d_3_.

### *CYP2D6* genotyping

To date, over 150 *CYP2D6* alleles have been identified and reported (www.PharmVar.org; CYP2D6 Allele Definition Table). These alleles can be classified into three categories based on their functions. In general, *CYP2D6* variant alleles encode for normal proteins, such as CYP2D6*1, *2, *27, and *35. Some alleles result in significantly decreased enzyme activity, such as CYP2D6*10, *17, *29, *36, *41, and *47. In addition, some non-functional alleles encode for inactive proteins, such as CYP2D6*3-6, and *14. The frequencies of each allele were observed to be significantly different among various geographic, racial, and ethnic populations. CYP2D6*10 is the most common genetic polymorphism among Asians, with a mutation frequency of 55.8% in Chinese. CYP2D6*14 is an allele unique to the Asian population [20], with a mutation frequency of 1.8% in Chinese [21]. The frequency of CYP2D6*2 is 12.05%. Therefore, this study ultimately selected the aforementioned three single nucleotide polymorphisms (SNPs), *i.e.*, CYP2D6*2, CYP2D6*10, and CYP2D6*14.

The combination of alleles is used to determine a patient’s diplotype. Each functional group is assigned an activity value ranging from 0 − 1 (*e.g*., 0 for no, 0.5 for decreased, and 1.0 for normal function, respectively). The CYP2D6 activity score (AS) is the sum of the values assigned to each allele. As shown in Supplemental Table 1, the patients were divided into the following *CYP2D6* genotype-defined categories according to the CPIC guideline [17]: children with an AS of 0 are poor metabolizers (PMs), those with a score of 0.5 are considered intermediate metabolizers (IMs), and those with a score of 1.0-2.0 represent extensive metabolizers (EMs). Of note, CYP2D6*10 allele seems to convey a reduction in activity across many substrates, which leads to a special recommendation for CYP2D6*10 containing diplotypes for atomoxetine. Hence, children and adolescents carrying CYP2D6*10/*10 alleles were divided into the IMs in this study.

For genotyping, DNA was extracted from venous blood samples of patients using a commercial blood DNA kit. These samples were left over from routine atomoxetine TDM testing. Genotyping of *CYP2D6* variant alleles was performed using Taqman-based real-time polymerase chain reaction assays, as described in detail elsewhere [22]. The resulting data were analyzed by GeneMarker and converted to AS and phenotype, as described above.

### Statistical analysis

All statistical analyses were performed with SPSS (Version 26.0, IBM SPSS Statistics, Armonk, NY, USA). GraphPad was used for graphical presentations (Version 9.0, GraphPad Software, San Diego, CA, USA). Descriptive data were presented as number (n) and percentage (%) for categorical variables, but median and interquartile range for continuous variables. Categorical variables were described using numbers and percentages (%). To compare the differences in clinical efficacy and adverse effects, we performed univariate analysis using the chi-square test (for categorical variables) or Fisher exact test. The linear mixed model analyses (using random intercept and the restricted maximum likelihood model) were used to allow for the inclusion of multiple samples per patient with age, sex, dosage form, and CYP2D6 phenotype. The Kruskal-Wallis test was utilized to compare dose-corrected concentrations (*C*/D) among multiple groups. The optimal cut-off values for efficacy were calculated by using the receiver operating characteristic (ROC) curve. *P* values less than 0.05 were considered statistically significant.

### Legal and ethical considerations

The study was conducted in accordance with the Helsinki Declaration. Medical data collection was approved by the Ethics Committee of the Children’s Hospital of Nanjing Medical University (Protocol number 202307002-1). No written informed consent was required for collecting and analyzing blood samples as part of the clinical routine TDM.

## Results

### Characteristics of study population

A total of 386 children and adolescents (326 boys, 60 girls) aged between 6 and 16 years were satisfied the criteria for inclusion and exclusion in this retrospective study. Of note, some children modified their medication regimen while receiving atomoxetine treatment. Consequently, 392 plasma atomoxetine concentrations from 303 subjects, 71 plasma atomoxetine concentrations from 62 subjects, and 53 plasma atomoxetine concentrations from 47 subjects on the *q.m*., *b.i.d*., and *q.n*. dosing schedule, respectively, were available for the further analysis (Table 1). Correspondingly, the average ages were 9.25, 9.67, and 9.08 years, respectively. Likewise, most enrolled children were males, accounting for 82.91%, 92.96%, and 90.57%, respectively. The most common co-morbidity among them was tic disorder, regardless of the dosing regimen group.

**Table 1:** Characteristics of study population.

	*q.m*.	*b.i.d*.	*q.n*.
Patients/Concentrations, *n*/N^a^	303/392	62/71	47/53
Sex, (*n*, %)			
Male	251, 82.84%	58, 93.55%	43, 91.49%
Female	52, 17.16%	4, 6.45%	4, 8.51%
Age (years)	9.25 (7.94–10.25)	9.67 (8.67–10.58)	9.08 (8.29–9.875)
Body weight (kg)	30 (25.80–36.33)	37.8 (30.00–43.50)	27.6 (24.90–32.95)
Dose (mg/day)	35 (25.00–40.00)	25 (10.00–25.00)	30 (25.00–35.00)
Concentrations (ng/mL)	322 (222–493)	216 (138–289)	36.3 (23–76)
Dose-adjusted serum concentrations (C/D, ng/mL/mg)	9.85 (6.25–14.08)	10.40 (7.32–15.3)^**b**^	1.09 (0.84–2.27)
Comorbidities (n, %)	47, 15.5%	9, 15.0%	6, 12.8%
Tic disorder	33, 10.9%	7, 11.3%	5, 10.6%
Epilepsy	8, 2.6%	NA	NA
Autism spectrum disorder	7, 2.3%	2, 3.3%	1, 2.1%
Conduct disorder	2, 0.6%	NA	NA
CYP2D6 phenotype, (*n*, %)			
EM	201, 66.34%	38, 61.29%	31, 65.96%
IM	101, 33.33%	24, 38.71%	16, 34.04%
PM^c^	1, 0.33%	NA	NA

^a^*n* values referred to corresponding number of cases; *N* values referred to number of concentration tests.

^b^Dose-adjusted plasma atomoxetine concentrations for *b.i.d*. dosing regimen were those doses taken prior to blood sampling.

^c^Only one CYP2D6 PM receiving *q.m*. dosing regimen was enrolled, who was not included in the subsequent analysis

Under the *q.m*., *b.i.d*., and *q.n*. dosing regimens, the median with upper and lower quartiles of plasma atomoxetine concentrations were 322 (222-493), 216 (138-289), and 36.3 (23-76) ng/mL, respectively. Impressively, the proportions of CYP2D6 EMs were 66.34%, 61.29%, and 65.96%, respectively. Of note, only one patient carrying PM phenotype (0.33%) was found under the *q.m*. dosing regimen, who was not included in the subsequent analysis. Finally, 515 plasma atomoxetine concentrations of 385 children (325 boys and 60 girls) with ADHD between 6 and 16 years of age in total were included for statistical analysis in this study.

### Comparison and multivariate analysis for *C*/D of atomoxetine

**Fig. 2:**
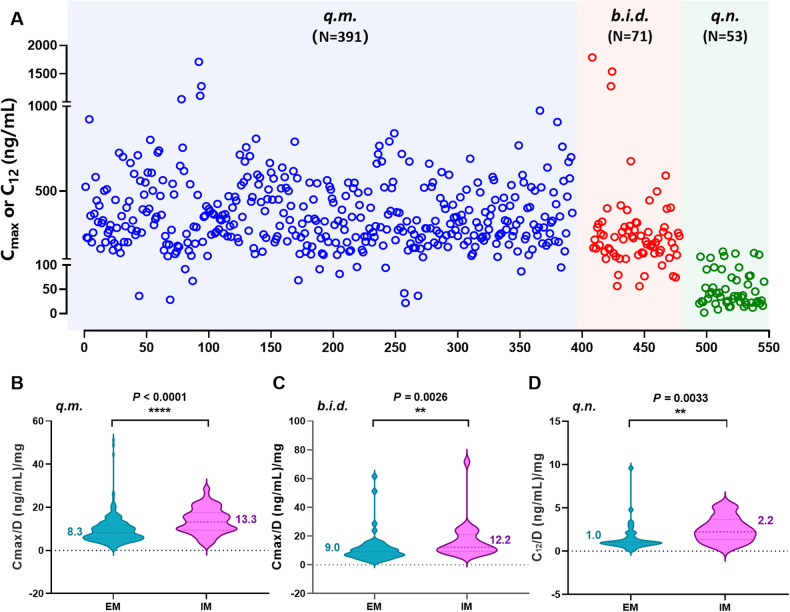
The exposure to atomoxetine. **A** The plasma concentration scatter plot of atomoxetine in the three dosing regimens. Blue, red and green circles indicate q.m., b.i.d. and q.n. regimens, respectively. **B–D** The corrected concentration differences among different CYP2D6 phenotypes under the three dosing regimens.

As shown in Fig. 2A, the exposure to atomoxetine varied markedly among individuals regardless of dosing regimens. In general, CYP2D6 IMs exhibited significantly higher atomoxetine *C*/D values than EMs under the three dosing regimens (Fig. 2B–D). The median *C*/D values in CYP2D6 IMs were 1.6, 1.4, and 2.2 times higher than those in EMs under *q.m*., *b.i.d*., and *q.n*. regimens, respectively. The linear mixed-effects models revealed that sex, BW, and CYP2D6 phenotype had significant impacts on *C*/D levels of atomoxetine (Table 2, *P* < 0.05). Specifically, the *C*/D values in males were lower by 1.79 units than in females (*β* = −1.79, *P* = 0.0322). The *C*/D values decreased by 0.12 units with every 1 kg increase in BW (*β* = -0.12, *P* = 0.0045). Notably, CYP2D6 phenotype was found to be the most crucial factor that affected the *C*/D levels, with those EMs experiencing a 4.34-unit decrease compared to those IMs (*β* = −4.34, *P* < 0.0001). Although the dosage form was found to be a significant factor affecting the atomoxetine *C*/D values in the *q.n*. regimen (*β* = −5.19, *P* < 0.0001), the statistical strength of this finding was relatively weak due to the limited data available (N = 2 in the oral solution group).

**Table 2:** Multivariate analysis for influencing factors on the daily-dose corrected atomoxetine concentration.

	Sex		Age		Body weight		Dosage form		Phenotype	
Dosing Regimen	*β*	*P*	*β*	*P*	*β*	*P*	*β*	*P*	*β*	*P*
***q.m.***	−1.79	**0.0322**	−0.40	0.1061	−0.12	**0.0045**	0.12	0.9389	−4.34	**<0.0001**
***b.i.d.***	0.04	0.9948	−0.39	0.7322	−0.20	0.2927	0.67	0.9534	−5.17	0.0858
***q.n.***	−0.99	0.1227	−0.07	0.7109	−0.03	0.4733	−5.19	**<0.0001**	−0.63	0.1238

Bold values represent statistically significant differences.

### Cutoff values for plasma atomoxetine concentrations required to achieve favorable clinical efficacy

**Fig. 3:**
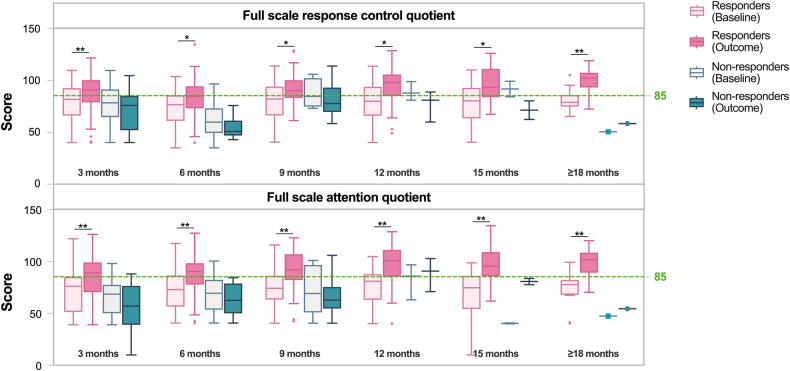
Score changes of IVA-CPT in children with ADHD during different followed up periods. Up: the score of full scale response control quotient. Down: the score of full scale attention quotient.

A total of 277 out of the original 385 eligible participants had both complete baseline and follow-up data on the IVA-CPT scale, and these individuals with 337 measurement data were analyzed to evaluate the efficacy of atomoxetine. Individuals were assigned to the aforementioned groups according to various follow-up periods, like 3-month, 6-month, 9-12-month, and over 15-month. As shown in Supplemental Table 3, during the 3-month follow-up, the six baseline quotient scores of IVA-CPT were similar between responders and non-responders, and the overall responsive rate of atomoxetine was 80.7%. However, the baseline scores of non-responders in full-scale response control quotient during the 6-month period, the auditory attention quotient during the 9-12-month period, and the full-scale attention quotient over the 15-month period were significantly lower than those of responders. But there were no differences in the baseline scores of the other IVA-CPT scores. Additionally, the overall responsive rate of atomoxetine was 86.5% in the 6-month follow-up period, 88.9% in the 9-12 month period, and 93.6% over the 15-month period. The changes of full-scale response control quotient and full scale attention quotient in detail are shown in Fig. 3.

**Fig. 4:**
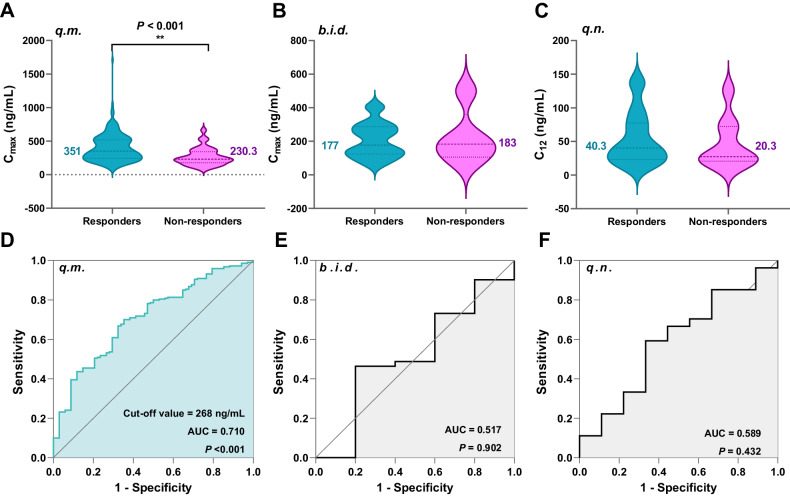
Plasma concentration differences and cut-off value between clinical efficacy of atomoxetine in children with ADHD. **A–C** The concentration differences between responders and non-responders under the three dosing regimens. **D–F** The cut-off values between responders and non-responders under the three dosing regimens.

Interestingly, it was also observed that plasma atomoxetine concentrations were significantly higher in children receiving *q.m*. regimen who exhibited improved clinical efficacy compared to those with insignificant improvement (Fig. 4A). We performed further cut-off values analysis under the three regimens using ROC curves. Impressively, individuals exhibited a more effective response to atomoxetine at a concentration above 268 ng/mL (Fig. 4D, ROC AUC = 0.710, *P* < 0.001) under *q.m*. dosing regimen. As expected, CYP2D6 IMs demonstrated a better clinical response than those EMs due to the higher drug exposure under *q.m*. dosing regimen (Table 3). However, statistically significant differences of concentrations (Fig. 4B, C), cut-off values (Fig. 4E-F), and clinical response were not found under the other two dosing regimens, possibly due to the small sample size.

**Table 3:** Clinical efficacy differences in enrolled children with different CYP2D6 phenotype under the three dosing regimens.

Dosing Regimen	Phenotype	Good/Poor Efficiency (N*, %)	*P* value	Concentration (Median, ng/mL)
				Good/Poor Efficiency
Overall	EM	178 (83.18)/36 (16.82)	0.1042	254/219.5
	IM	110 (90.16)/12 (9.84)		400/190
*q.m.*	EM	133 (85.71)/28 (14.29)	**0.0132**	295/242.5
	IM	87 (93.55)/6 (6.45)		493/198.5
*b.i.d.*	EM	27 (93.1)/2 (6.9)	0.3429	144/105.2
	IM	14 (82.35)/3 (17.65)		274.5/221
*q.n.*	EM	18 (75)/6 (25)	>0.9999	35.65/26.4
	IM	9 (75)/3 (25)		77.4/126

*N values referred to corresponding number of concentration values.

### Adverse reactions of atomoxetine treatment

As shown in Table 4, when not considering *CYP2D6* genotypes and phenotypes, decreased appetite was the most common adverse reactions of atomoxetine, with incidence rates of 32.99%, 26.76%, and 22.64% under the *q.m*., *b.i.d*., and *q.n*. dosing regimens, respectively (*P* < 0.001). In comparison, CYP2D6 IMs experienced a higher risk of loss of weight and irritability than those EMs under *q.m*. dosing regimen (14.39 *vs* 5.02%, 15.15 *vs* 5.79%, respectively). Similarly, the CYP2D6 IMs showed a higher risk of decreased appetite, being easy to wake, and difficulty falling asleep than the EMs under *b.i.d*. dosing regimen (48.00 *vs* 15.22%, 16.00 *vs* 2.17%, 16.00 *vs* 2.17%, respectively).

**Table 4:** The profile of adverse reactions in enrolled children with different CYP2D6 phenotype under three dosing regimens.

	*q.m*. (N* = 391)			*b.i.d*. (*N* = 71)			*q.n*. (*N* = 53)		
	EM (*N* = 259, %)	IM (*N* = 132, %)	*P* value^*a*^	EM (*N* = 46, %)	IM (*N* = 25, %)	*P* value^*a*^	EM (*N* = 37, %)	IM (*N* = 16, %)	*P* value^*a*^
Decreased appetite	87 (33.59)	42 (31.82)	0.7245	7 (15.22)	12 (48.00)	**0.0029**	9 (24.32)	3 (18.75)	0.6562
Loss of weight	13 (5.02)	19 (14.39)	**0.0014**	2 (4.35)	2 (8.00)	0.5238	1 (2.70)	1 (6.25)	0.5338
Abdominal discomfort	10 (3.86)	1 (0.76)	0.0793	NA	NA	>0.9999	1 (2.70)	1 (6.25)	0.5338
Nausea and vomiting	20 (7.72)	6 (4.55)	0.2332	1 (2.17)	1 (4.00)	0.6569	NA	2 (12.50)	0.0871
Constipation	5 (1.93)	2 (1.52)	0.7696	NA	2 (8.00)	0.1207	NA	1 (6.25)	0.3019
Drowsiness	5 (1.93)	7 (5.30)	0.0675	1 (2.17)	3 (12.00)	0.0863	1 (2.70)	NA	>0.9999
Irritability	15 (5.79)	20 (15.15)	**0.0022**	5 (10.87)	2 (8.00)	0.6985	5 (13.51)	2 (12.50)	0.9203
Depression	4 (1.54)	4 (3.03)	0.3264	1 (2.17)	NA	>0.9999	1 (2.70)	NA	>0.9999
Dizziness and headache	9 (3.47)	7 (5.30)	0.3882	NA	1 (4.00)	0.3521	2 (5.41)	NA	>0.9999
Easy to wake	4 (1.54)	1 (0.76)	0.5126	1 (2.17)	4 (16.00)	**0.0296**	1 (2.70)	2 (12.50)	0.1565
Difficulty falling asleep	12 (4.63)	2 (1.52)	0.1166	1 (2.17)	4 (16.00)	**0.0296**	1 (2.70)	1 (6.25)	0.5338

^*^N values referred to the corresponding number of concentration values; ^a^referred to Chi-square test or Fisher exact test. Bold values represent statistically significant differences.

We conducted a further analysis to examine the potential relationship between concentrations and adverse effects of atomoxetine (Table 5). In the case of CYP2D6 IMs under *q.m*. dosing regimen, those who suffered from gastrointestinal adverse reactions had significantly higher atomoxetine concentrations than those who did not experience any adverse reactions (510.0 *vs* 386.0 ng/mL, *P* = 0.0411). Contradictorily, plasma atomoxetine concentrations were slightly decreased in CYP2D6 EM cases under *b.i.d*. dosing regimens who developed gastrointestinal adverse reactions than those who did not (151.5 vs. 175.9 ng/mL), while they had significantly lower atomoxetine concentrations in cases of nervous system adverse reactions (90.9 vs. 175.9 ng/mL).

**Table 5:** The association between adverse reactions and plasma atomoxetine concentrations (ng/mL) in enrolled children with different CYP2D6 phenotype under three dosing regimens.

	*q.m*. (N* = 391)		*b.i.d*. (*N* = 71)		*q.n*. (*N* = 53)	
	EM (*N* = 259)	IM (*N* = 132)	EM (*N* = 46)	IM (*N* = 25)	EM (*N* = 37)	IM (*N* = 16)
No-adverse reactions	286.0 (214.2–407.0)	386.0 (269.2–550.3)	175.9 (133.5–246.5)	306.0 (189.5–396.0)	35.65 (23.20–66.93)	73.15 (36.65–117.3)
Gastrointestinal adverse reactions	273.0 (193.2–411.2)	510.0 (323.5–639.0)	151.5 (107.5–1031.5)	275.0 (222.5–522.2)	25.50 (21.60–60.10)	59.70 (39.475–108.75)
Nervous system adverse reactions	243.5 (171.2–335.2)	501.0 (317.2–618.7)	90.9 (62.0–175.25)	260.5 (222.5–421.7)	23.80 (20.40–30.40)	65.90 (40.3–132.0)
*P* value^a^	0.9948	**0.0411**	0.4234	0.4493	0.9381	0.9358
*P* value^b^	0.3221	0.1396	**0.0115**	0.8030	0.6249	0.9821

^*^N values referred to corresponding number of concentration values.

Bold values represent statistically significant differences.

One way ANOVA analysis compares the mean of each column with the mean of a control column.

^a^Gastrointestinal adverse reactions *vs* No-adverse reactions.

^b^Nervous system adverse reactions *vs* No-adverse reactions.

## Discussion

**Fig. 5:**
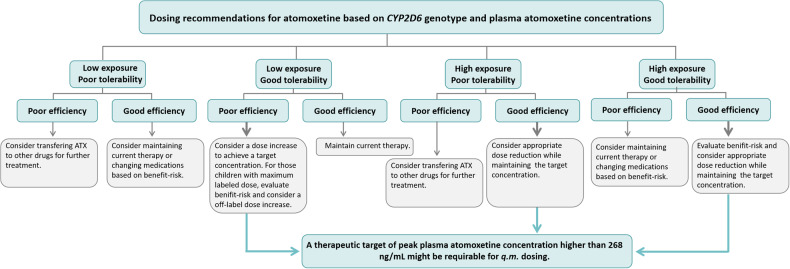
Dosing recommendations for atomoxetine based on *CYP2D6* genotype and plasma atomoxetine concentrations. Initiating with a dose of 0.5 mg/(kg·d) and adjusted to 1.2-1.4 mg/(kg·d) within 2–4 weeks of administration, and the maximum dose did not exceed 1.4 mg/(kg·d). Cut-off values of plasma atomoxetine concentrations were obtained at 1.5 to 2 h administration under the *q.m*. dosing.

To be honest, this study is the first retrospective clinical research exploring the individualized medication of atomoxetine by combining TDM with *CYP2D6* genotype in Chinese pediatric patients with ADHD. As will be discussed in more detail below, the results of our study suggest one possible strategy to tailor the nonstimulant to a patient’s individual needs (Fig. 5).

The critical question in this study is identifying the minimum systemic exposure that is associated with a high probability of optimal clinical response and the dose (and dosing regimen) that needs to be prescribed for individual children given their *CYP2D6* genotype and other factors contributing to the dose-exposure relationship.

Indeed, plasma peak concentrations of exposure check ranging from 200 to 1,000 ng/mL for adults are commonly regarded as “therapeutic”, measured 60–90 min after dosing of 1.2 mg/(kg·d) atomoxetine [16, 23]. Of note, in the 2019 CPIC guideline for children and adults, the peak concentration of atomoxetine is recommended as follows: If < 200 ng/mL, consider a proportional increase in dose to approach 400 ng/mL [17]. Thereafter, Sugimoto et al. revealed a trough threshold of 64.6 ng/mL in plasma, sampled approximately 12 h after the last dose, for pediatric patients in a non-randomized prospective interventional study [24]. In a very recent naturalistic study, Ruppert et al. proposed a preliminary therapeutic reference range, *i.e.*, 100 to 400 ng/mL, measured at the time point of 90 min after atomoxetine intake for children and adolescents with ADHD [25].

In our study, as expected, when considering dosing regimen, CYP2D6 IM patients generally experienced higher drug exposure than EM patients to achieve a good clinical response. Specifically, in the case of *q.m*. dosing, the CYP2D6 IMs’ median plasma atomoxetine concentration was 1.64 times higher than that of the EMs (448 vs. 274 ng/mL) (Supplemental Fig. 1). Moreover, in this dosing regimen, IMs were more likely to experience adverse reactions in the central nervous system and gastrointestinal system when plasma atomoxetine concentrations reached 501 and 510 ng/mL, respectively (Table 5).

Indeed, as of now, there is no well-defined therapeutic reference range with a lower and upper threshold of atomoxetine for children and adolescents. For some specific individuals, a concentration beyond the routinely recommended range may be warranted to achieve a favorable therapy response [26]. In fact, the issue of the potential for a subpopulation of patients who did not respond to atomoxetine to exist, while the potential for some other patients to respond to very low plasma atomoxetine concentrations did occur. Perhaps there is no rational explanation for this phenomenon from a pharmacokinetic perspective alone. Additionally, the mechanisms underlying atomoxetine-related adverse reactions are complex, and there is currently insufficient evidence to support the correlation between plasma concentration and adverse reactions.

Collectively, proposing a recommended range of minimum plasma concentrations required to achieve a good therapeutic effect may be more practical, whereas proposing reference targets that match the occurrence of adverse reactions may be much more difficult. Assuredly, *q.m*. dosing is the most commonly used dosing regimen for Chinese children and therefore the following therapeutic targets could be suggested for a requisite peak plasma atomoxetine concentration higher than 268 ng/mL (Fig. 5).

As expected, the *CYP2D6* genotype was the most significant influencing factor for the systemic exposure to atomoxetine, especially for *q.m*. dosing (Table 2). Due to the highly polymorphic nature, over 150 allelic variants of *CYP2D6* have been identified to date (https://www.pharmvar.org/gene/CYP2D6; Access time 6/17/2023). The most commonly reported alleles for east Asian are CYP2D6*2, *10, and *14 (CYP2D6 frequency table, https://cpicpgx.org/guidelines/cpic-guideline-for-atomoxetine-based-on-cyp2d6-genotype/) and the alleles are further categorized into normal function, decreased function, and no function, respectively. The genotypes were translated into a standardized phenotype classification system, *i.e.*, PM, IM, and EM, respectively, as shown in Supplemental Table 1.

In addition, from a developmental pharmacology perspective, the CYP2D6 protein expression was significantly increased in the first week after childbirth and reached adult maturity levels at several months of age [27]. Thus, for children aged 6 years and older, the impact of ontogeny on CYP2D6 activity may become less significant, with genotype being the key determinant. In this sense, the impact of *CYP2D6* genotype and phenotype on the function is comparable between 6-year-old children and adults.

In our study, the plasma atomoxetine concentrations in CYP2D6 IMs corrected for dose were significantly higher than those in EMs, ranging from 1.36 to 2.20 times higher in the former compared to the latter (Fig. 2B–D). Indeed, in earlier studies of healthy volunteers from China [28] and Japan [29], it was found that CYP2D6*10/*10 carriers who were classified as IMs had significantly higher plasma atomoxetine concentrations than individuals with other genotypes who were defined as EMs based on their systemic exposure levels. Comparable results were observed in healthy individuals from Korea [30]. Compared to the CYP2D6*wt/*wt group, the CYP2D6*10/*10, and CYP2D6*wt/*10 groups exhibited 1.74- and 1.15-fold higher *C*_max_, respectively, as well as 3.40- and 1.33-fold higher AUC, and 69.7% and 24.6% lower *CL/F*, respectively. Three years later, this Korean team repeated their previous findings in an expanded population [31].

Similar studies are very uncommon in pediatric populations. The study by Brown *et al*. showed that the peak concentration and time to peak concentration were significantly elevated in CYP2D6 PMs compared to the IMs, EMs1 (1 functional allele), and EMs2 (2 functional alleles) groups. There was a 30-fold difference in the AUC among ADHD patients with different genotypes. The oral *CL/F* of atomoxetine was also significantly correlated with *CYP2D6* genotype, with a *CL/F* of 6.0% in the PM group compared to the EMs2 group. Additionally, the *t*_1/2_ in the PM group was 2.9 times longer than in the IM group, and 5.4 to 5.9 times longer than in the EMs1 and EMs2 groups, respectively [12].

Additionally, through multiple-factor analysis, we revealed that both sex and BW significantly influenced the *C*/D values in children who received *q.m*. dosing (Table 2). Indeed, some studies in adults reported thus far have claimed that CYP2D6 activity is not related to biological sex [32], but other studies have yielded conflicting results on the effects of sex on the CYP2D6 activity [33].

Another relevant observation of our study was that the clinical effectiveness and adverse reactions of the atomoxetine therapy were closely related to *CYP2D6* genotype, which might due to its indirect influence on systemic exposure to atomoxetine. We revealed that there was no difference in clinical effectiveness among the three administration regimens if the influence of *CYP2D6* genotype was not considered. However, after re-grouping analysis based on the *CYP2D6* genotype, the differences became apparent and the effectiveness of CYP2D6 IMs was significantly better than that of EMs (Table 3). Indeed, if prescribed the same absolute or BW-based doses of atomoxetine as CYP2D6 EMs, IMs would be expected to have higher systemic exposure and presumably a higher probability of clinical response, assuming that an exposure-response relationship exists.

There have been some studies reporting differences in the clinical efficacy of atomoxetine under different dosing regimens. Indeed, some studies revealed that the clinical efficacy of *q.m*. and *b.i.d*. dosing seemed to be consistent [34]. Other reports also showed that both *q.m*. and *q.n*. dosing could significantly reduce ADHD core symptoms, and their efficacy was similar [35]. However, intriguingly, for certain efficacy indicators, *q.m*. dosing was preferred over *q.n*. dosing [35], which was in line with an early report performed by Kelsey et al. [36]. They revealed that *q.m*. dosing significantly improved ADHD core symptoms, and its efficacy could last until the next morning [36].

Nevertheless, those above-mentioned studies did not take into account the potential impact of *CYP2D6* genotypes. Indeed, in an early study, no significant difference in the treatment effect, as measured by ADHD rating scale end point values, was observed between CYP2D6 EMs and PMs, in any tested cohorts [37]. Very recently, the CPIC guideline summarized that individuals who are CYP2D6 PMs have higher chances of experiencing positive treatment responses compared to non-PMs, likely attributable to the increased exposure to atomoxetine in the PMs. Furthermore, PMs exhibited more significant improvements in ADHD symptoms in comparison to non-PMs. Conversely, CYP2D6 non-PMs had a higher likelihood of discontinuing atomoxetine therapy due to inefficacy in comparison to PMs [17].

In addition, it seems that the clinical efficacy of atomoxetine cannot be conclusively determined for which age group of children it works best. A meta-analysis suggested that the treatment with atomoxetine had a greater overall response in improving ADHD symptoms of children aged 6-7 years as compared to those aged 8-12 years [38]. However, extrapolation analysis of some clinical trial data revealed that in 5-year-old patients with ADHD, atomoxetine might improve ADHD symptoms, but possibly to a lesser extent than in older children [39]. Likewise, results of another meta-analysis of these short-term, controlled, multi-site studies observed a similar responsive rate to atomoxetine therapy between children (6–11 years) and adolescents (12–17 years) with ADHD [40], which was in line with our findings (Supplemental Table 2).

Next, we further assessed the potential association between plasma atomoxetine concentration, based on the *CYP2D6* genotypes, and the adverse reactions. First, we found in our study that, when not considering *CYP2D6* genotypes, the incidence of decreased appetite was significantly higher with *q.m*. dosing compared to the *b.i.d*. dosing (Table 4). Coincidentally, early studies found that, within the first 2 weeks of treatment, the incidence of decreased appetite and drowsiness in children was significantly higher with *q.m*. dosing compared to the *b.i.d*. dosing, while the incidence of headache was lower. However, by the 8th week, more adverse reactions were reported with the latter dosing regimen [41]. Furthermore, a study in adults showed that the likelihood of nausea was significantly reduced with the *b.i.d*. dosing regimen, while the likelihood of constipation in patients receiving *q.m*. dosing showed a decreasing trend [42].

Second, the *CYP2D6* genotype appeared to play an important role in both safety and tolerability indirectly through its influence on systemic exposure. The CPIC guideline tells us that the likelihood of side effects is also reported to be higher in CYP2D6 PMs compared to non-PMs, which is likely due to increased exposure to atomoxetine itself in the PMs [17]. For example, compared to CYP2D6 EMs, PMs are more likely to an increase in heart rate and diastolic blood pressure, a decrease in weight gain, as well as adverse reactions such as decreased appetite [23]. However, some studies found that *CYP2D6* genotypes were not associated with the safety and tolerability of atomoxetine treatment [28]. In our study, in CYP2D6 EMs, *q.m*. dosing was more likely to cause decreased appetite compared to the other two dosing regimens, while in IMs, the incidence of decreased appetite was lowest with *q.n*. dosing (Table 4). Of note, in our study, there was only one PM patient, which could not be included in the statistical analysis or evaluated for tolerability.

Impressively, for the first time, we found that some adverse reactions occurred in a concentration-dependent manner. For example, in CYP2D6 IMs receiving *q.m*. dosing or EMs receiving the *b.i.d*. dosing, the incidence of gastrointestinal and neurological adverse reactions was significantly different at the plasma atomoxetine concentrations when no adverse reactions occurred (Table 5).

One more concern needs to be further discussed. Comorbidity and drug-drug interactions might affect the plasma atomoxetine concentration. Assuredly, in our study, we revealed that stratifying patients according to CYP2D6 phenotype showed that comorbidity did not affect the plasma atomoxetine concentrations after dose adjustment (data not shown). There were rare cases of co-administration with other drugs among the enrolled children receiving atomoxetine treatment, so the potential impact of drug-drug interactions can be ignored. In addition, our study subjects also did not involve the combined use of traditional Chinese herbs, so there was no need to concern potential herb-drug interactions either. Impressively, Belle et al. found that inhibition of CYP2D6 by paroxetine markedly affected the atomoxetine disposition in healthy adults, resulting in pharmacokinetics similar to PMs of CYP2D6 substrates [43], which was line with a very recent study reported by Jung et al. [44]. Therefore, the possibility of changes in systemic exposure to atomoxetine should be given adequate attention when co-administered with other medications, particularly CYP2D6 inhibitors, due to comorbidity treatment.

One of the major strengths of the present study was our ability to assess the treatment response and side effects of atomoxetine therapy in Chinese children with ADHD by integrating TDM and *CYP2D6* genetic testing. Of note, 515 plasma atomoxetine concentrations of 385 children with ADHD between 6 and 16 years of age were included and analyzed in our study. This is the first study conducted in Chinese children to explore the potential implementation of individualized dosing based on *CYP2D6* genotypes and plasma atomoxetine concentrations under different dosing regimens, and it is also the largest retrospective study reported in the world. Indeed, we performed stratified analysis of clinical efficacy and adverse reactions based on *CYP2D6* genotype for the three commonly used clinical dosing regimens, attempting to propose recommended reference ranges for monitoring plasma atomoxetine concentration for each scenario (Fig. 5).

However, our study has several limitations due to its retrospective nature. First, the number of participants enrolled in the *b.i.d*. dosing (*n* = 62) and *q.n*. dosing (*n* = 47) regimens was relatively small, and after stratifying by *CYP2D6* genotype, much fewer patients were allocated to the each group, which may weaken the statistical power of our findings. Second, *CYP2D6* genotyping was only limited to *2, *10, and *14. Although these might be the most common expected alleles in Chinese patients, one cannot exclude the possibility of some rare alleles contributing to miss assignment of predicted CYP2D6 phenotype, especially given that comparison of clinical response between EMs and IMs was considered in this study. In addition to CYP2D6, variations in atomoxetine response have also been examined with its metabolizing enzyme, like CYP2C19 [45, 46] and its pharmacodynamic target, like SLC6A2 [37, 47]. The potential effects of these genetic variations were not incorporated in our study either. The third limitation of this study was that, in children with IVA-CPT baseline scores, their follow-up periods varied. During the efficacy assessment, we grouped those with the same follow-up time for statistical analysis, resulting in varying numbers of children with different follow-up periods and compositions included, which might contribute to heterogeneity in the data analysis. Lastly, 4-OH atomoxetine is an active metabolite but is rapidly metabolized in the body and lacks recommended reference ranges. Therefore, we did not routinely test for it in the TDM of atomoxetine.

To summarize, this retrospective study in children with ADHD achieved the following key findings: (1) the *CYP2D6* genotype was identified as the most significant factor affecting the systemic exposure to atomoxetine; (2) the clinical efficacy and tolerability of atomoxetine therapy were associated with the *CYP2D6* genotype; (3) the results of this study provided evidence for recommending specific treatment strategies based on *CYP2D6* genotype and plasma atomoxetine concentration monitoring. A peak plasma atomoxetine concentration higher than 268 ng/mL might be requisite for *q.m*. dosing. To validate and reinforce these initial findings, it is necessary to collect further data in controlled studies with a larger sample size.

### Supplementary information

supplemental documents

## Data Availability

The data that support the findings of this study are available from the corresponding author upon reasonable request.
