# Supplementary material for: Individualized atomoxetine response and tolerability in children with ADHD receiving different dosage regimens: the need for CYP2D6 genotyping and therapeutic drug monitoring to dance together
Source: Transl Psychiatry. 2024 Mar 19;14:151. doi: 10.1038/s41398-024-02859-2 (PMC10951231; doi:10.1038/s41398-024-02859-2)

**Individualized atomoxetine response and tolerability in children with ADHD receiving different dosage regimens: The need for *CYP2D6* genotyping and therapeutic drug monitoring to dance together**

Hong-Li Guo^1,†^, Dan-Dan Wu^2,†^, Di Fu^3,‡^, Yue Li^1^, Jie Wang^1^, Yuan-Yuan Zhang^1^, Wei-Jun Wang^3,‡^, Jian Huang^1^, Wei-Rong Fang^3^, Jing Xu^1^, Ya-Hui Hu^1,*^, Qian-Qi Liu^2, *^, Feng Chen^1,*^

^1^ Department of Pharmacy, Children’s Hospital of Nanjing Medical University, Nanjing, China

^2^ Department of Children Health Care, Children’s Hospital of Nanjing Medical University, China

^3^ School of Basic Medicine and Clinical Pharmacy, China Pharmaceutical University, Nanjing, China

^†^These authors contributed equally to this work.

^‡^Visiting graduate student from China Pharmaceutical University.

***Corresponding authors:**

Email addresses: [huyahui324@163.com](mailto:huyahui324@163.com) (Ya-Hui Hu), [18951769617@163.com](mailto:18951769617@163.com) (Qian-Qi Liu) and [cy.chen508@gmail.com](mailto:cy.chen508@gmail.com) (Feng Chen).

**Supplementary Data**

Supplemental Table 1 CYP2D6 genotyping and assignment of likely CYP2D6 phenotypes based on diplotypes

| **Alleles** | | | **Diplotypes** | **Activity Score** | **Phenotype** |
| --- | --- | --- | --- | --- | --- |
| **100C>T** | **1758G>A** | **2850C>T** |  |  |  |
| CC | GG | CC | *1/*1 | 1-2 | EM |
|  |  | CT | *1/*2 | 1-2 | EM |
|  |  | TT | *2/*2 | 1-2 | EM |
|  | GA | CC | *14/*1 | 1 | IM |
|  |  | CT | *14/*2 | 1 | IM |
|  |  | TT | *14/*2 | 1 | IM |
|  | AA | CC | *14/*14 | 0 | PM |
|  |  | CT | *14/*14 | 0 | PM |
|  |  | TT | *14/*14 | 0 | PM |
| TT | GG | CC | *10/*10 | 1 | IM |
|  |  | CT | *10/*10 | 1 | IM |
|  |  | TT | *10/*10 | 1 | IM |
|  |  | **ND*** | *4 | 0 | PM |
|  | GA | CC | *14/*10 | 0.5 | IM |
|  |  | CT | *14/*10 | 0.5 | IM |
|  |  | TT | *14/*10 | 0.5 | IM |
|  | AA | CC | *14/*14 | 0 | PM |
|  |  | CT | *14/*14 | 0 | PM |
|  |  | TT | *14/*14 | 0 | PM |
| CT | GG | CC | *10/*1 | 1.5 | EM |
|  |  | CT | *10/*2 | 1.5 | EM |
|  |  | TT | *10/*2 | 1.5 | EM |
|  | GA | CC | *14/*1 | 1 | IM |
|  |  | CT | *14/*1 | 1 | IM |
|  |  | TT | *14/*1 | 1 | IM |
|  | AA | CC | *14/*14 | 0 | PM |
|  |  | CT | *14/*14 | 0 | PM |
|  |  | TT | *14/*14 | 0 | PM |
| ND* | ND | ND | *5 |  | PM |

***,** ND denotes no result was detected, indicating a missing locus.

*Abbreviations*: EM, Extensive metabolizers; IM, Intermediate metabolizers; PM, Poor metabolizers.

Supplemental Table 2 Clinical efficacy of atomoxetine under different dosing regimens in two age categories (N* = 336).

| **Dosing Regimen** | **Age** | **Good/Poor Efficiency（N，%）** | **P** |
| --- | --- | --- | --- |
| ***q.m.*** | School-age | 207 (86.25)/33 (13.75) | 0.701 |
|  | Adolescence | 13 (92.86)/1 (7.14) |  |
| ***b.i.d.*** | School-age | 38 (88.37)/5 (11.63) | >0.9999 |
|  | Adolescence | 3/0 |  |
| ***q.n.*** | School-age | 26 (74.29)/9 (25.71) | >0.9999 |
|  | Adolescence | 1/0 |  |

N values referred to corresponding number of concentration values.

Supplemental Table 3 IVA-CPT scores between atomoxetine responders and non-responders during different follow-up periods

| **Items** |  | **3-month** | | | **6-month** | | | **9 - 12 months** | | | **≥15 months** | | |
| --- | --- | --- | --- | --- | --- | --- | --- | --- | --- | --- | --- | --- | --- |
|  |  | **Responders** | **Non-responders** | ***P* value** | **Responders** | **Non-responders** | ***P* value** | **Responders** | **Non-responders** | ***P* value** | **Responders** | **Non-responders** | ***P* value** |
|  |  | **(N = 109)** | **(N = 26)** |  | **(N = 64)** | **(N = 10)** |  | **(N = 72)** | **(N = 9)** |  | **(N = 44)** | **(N = 3)** |  |
| **Auditory response control quotient** | Baseline | 83 (68-94) | 83 (70-93) | 0.955 | 86 (73-94) | 75 (61-87) | 0.244 | 83 (70-94) | 84 (78-106) | 0.332 | 83 (71-89) | 69 | - |
|  | Outcome | 94 (83-104) | 80 (57-92) | **0.001*** | 94 (83-103) | 66 (61-71) | **<0.001**** | 98 (88-107) | 78 (66-93) | **0.003*** | 101 (88-111) | 82 (75-89) | 0.112 |
|  | *P* value | **<0.001**** | 0.609 |  | **0.006*** | 0.204 |  | **<0.001**** | 0.281 |  | **<0.001**** | - |  |
| **Visual response control quotient** | Baseline | 84 (64.5-95) | 82 (70-104) | 0.582 | 83 (72.5-93.5) | 73 (53.5-77.5) | 0.051 | 85 (66-95) | 92 (81-98) | 0.29 | 84 (67.5-90.5) | 106 | - |
|  | Outcome | 92 (77-101) | 72 (60-91) | **<0.001**** | 88 (77-101) | 55 (53-74) | **<0.001**** | 93 (83-102) | 83 (72-105) | 0.519 | 93 (86-105) | 75 (60-90) | 0.135 |
|  | *P* value | **<0.001**** | 0.088 |  | 0.1 | 0.599 |  | **0.001*** | 0.716 |  | **<0.001**** | - |  |
| **Full scale response control quotient** | Baseline | 82 (66-93) | 79 (65-92) | 0.776 | 82 (66-91) | 65 (55-78) | **0.042*** | 81 (66-93.75) | 88 (76.5-100) | 0.259 | 79 (73-90) | 84 (50-99) | 0.825 |
|  | Outcome | 91 (79-101) | 76 (52-85) | **<0.001**** | 90 (78-100) | 56 (52-66) | **<0.001**** | 95 (84-104) | 78 (67-88) | **0.011*** | 99 (86-108) | 62 (58-62) | 0.003* |
|  | *P* value | **<0.001**** | 0.306 |  | **0.003*** | 0.271 |  | **0.001*** | 0.231 |  | **<0.001**** | 0.7 |  |
| **Auditory attention quotient** | Baseline | 80 (63-91) | 73 (66-79) | 0.115 | 79 (61.75-90.5) | 82 (55-94.5) | 0.974 | 86 (70-97) | 61 (57-80) | **0.017*** | 81 (71.5-95) | 50 | - |
|  | Outcome | 95 (74-102) | 71 (51-76) | **<0.001**** | 92 (78-101) | 62 (46-78) | **0.004*** | 99 (86-109) | 78 (53-100) | **0.023*** | 104 (88-112) | 85 (83-86) | 0.137 |
|  | *P* value | **<0.001**** | 0.342 |  | **<0.001**** | 0.354 |  | **<0.001**** | 0.351 |  | **<0.001**** | - |  |
| **Visual attention quotient** | Baseline | 77 (55-88) | 68 (54-85) | 0.398 | 77 (61.75-91.25) | 69 (41.5-76.5) | 0.231 | 75 (65-91) | 69 (62-84) | 0.594 | 75 (59.5-84.5) | 42 | - |
|  | Outcome | 89 (76-100) | 63 (53-82) | **<0.001**** | 90 (82-98) | 68 (59-79) | **<0.001**** | 97 (85-109) | 71 (60-99) | **0.008*** | 98 (86-110) | 78 (76-78) | 0.104 |
|  | *P* value | **<0.001**** | 0.554 |  | **<0.001**** | 0.867 |  | **<0.001**** | 0.516 |  | **<0.001**** | - |  |
| **Full scale attention quotient** | Baseline | 77 (53-86) | 70 (51-79) | 0.13 | 73 (56-86) | 69 (53-82) | 0.577 | 79 (63.25-87) | 76 (58-96) | 0.891 | 76 (57-84) | 41 (40-47) | **0.015*** |
|  | Outcome | 90 (72-100) | 58 (40-77) | **<0.001**** | 90 (77-98) | 62 (49-78) | **<0.001**** | 95.5 (85-108) | 65 (60-97) | **0.007*** | 97.5 (89-108.8) | 78 (54-78) | **0.013*** |
|  | *P* value | **<0.001**** | 0.208 |  | **<0.001**** | 0.591 |  | **<0.001**** | >0.999 |  | **<0.001**** | 0.1 |  |

Statistically significant in analysis of covariance **P <0.001, *P <0.05. N values referred to corresponding number of concentration values. Bold values represent statistically significant differences

Supplemental Figure 1 The concentration differences among different CYP2D6 phenotypes under the three dosing regimens.


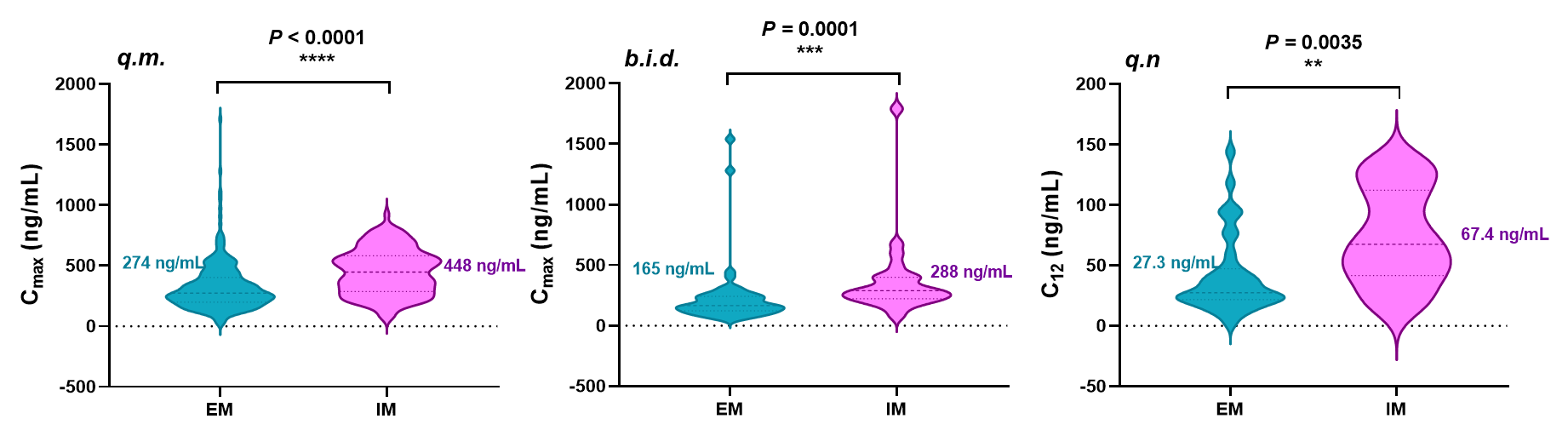

Supplement: Supplementary file 1 — supplemental documents [file 41398_2024_2859_MOESM1_ESM.docx]
